# Effect of Silymarin as an Adjunct Therapy in Combination with Sofosbuvir and Ribavirin in Hepatitis C Patients: A Miniature Clinical Trial

**DOI:** 10.1155/2022/9199190

**Published:** 2022-02-02

**Authors:** Sarfraz Ahmed, Najeeb Ullah, Sadia Parveen, Ifra Javed, Nur Asyilla Che Jalil, Mogana Das Murtey, Irfan Shahzad Sheikh, Shahroz Khan, Suvash Chandra Ojha, Ke Chen

**Affiliations:** ^1^Department of Basic Sciences, University of Veterinary and Animal Sciences Lahore, 51600 Narowal, Pakistan; ^2^Department of Biochemistry, Bahauddin Zakariya University, Multan 60800, Pakistan; ^3^Departments of Pathology, School of Medical Sciences, Universiti Sains Malaysia, 16150 Kubang Kerian, Kelantan, Malaysia; ^4^Basic Sciences and Oral Biology Unit, School of Dental Sciences, Health Campus, Universiti Sains Malaysia, 16150 Kubang Kerian, Kelantan, Malaysia; ^5^Centre for Advanced Studies Vaccinology and Biotechnology, University of Balochistan, Quetta, Pakistan; ^6^Noor Nursing College, Swari, 19290 Buner, Pakistan; ^7^Department of Infectious Diseases, The Affiliated Hospital of Southwest Medical University, 646000 Luzhou, China; ^8^Southwest Medical University, Jiangyang District, Luzhou, 646000 Sichuan, China

## Abstract

Silymarin is proclaimed to be a blend of flavonolignans or phytochemicals. An era of new generation of direct-acting antivirals (DAAs) has commenced to have facet effect in swaying of the hepatitis C virus (HCV). Nonetheless, this therapy has serious side effects that jeopardize its efficacy. This study is aimed at probing the effects of ribavirin (RBV) and sofosbuvir (SOF) along with silymarin as an adjunct therapy on hematological parameters and markers of obscured oxidative stress. The effect of DAAs along with silymarin was also examined on variable sex hormone level and liver function markers such as alanine aminotransferase (ALT), aspartate transaminase (AST), alkaline phosphatase (ALP), and bilirubin. The study was followed to determine viral load and viral genotypes. A total of 30 patients were randomly divided into two equal groups comprising the control group (*n* = 15) and treatment group (*n* = 15). The control group was solely administered with DAAs (SOF and RBV; 400 mg/800 mg each/day). Conversely, the treatment group was dispensed with DAAs, but with adjunct therapy of silymarin (400 mg/day) along with DAAs (400/800 mg/day) over period of 8 weeks. Sampling of blood was performed at pre- and posttreatment levels for the evaluation of different propound parameters. Our data showed that silymarin adjunct therapy enhances the efficiency of DAAs. A decrease in menace level of liver markers such as ALT, ALP, AST, and bilirubin was observed (*p* > 0.05). The adjunct therapy concurrently also demonstrated an ameliorative effect on hematological indices and oxidative markers, for instance, SOD, TAS, GSH, GSSG, and MDA (*p* < 0.05), diminishing latent viral load. The silymarin administration was also found to revamp the fluster level of sex hormones. Our outcomes provide evidence that systematic administration of silymarin effectively remits deviant levels of hematological, serological, hormonal, and antioxidant markers. This demonstrates a possibly unique role of silymarin in mitigating hepatitis C.

## 1. Introduction

Hepatitis C is a pathological disorder caused by the hepatitis C virus (HCV) infection. Approximately, more than 185 million people around the globe have been infected by HCV [1]. Hepatitis is presumed to be a huge economic and health problem worldwide, especially in developing countries [2]. It is estimated that each year, 1.4 million deaths befall globally owing to viral hepatitis over other infectious diseases. Recent estimation demonstrated that HCV infection becomes a provocative problem faced by humanity for decades [3].

HCV contagion entails chronic liver disease and a significant ratio of patients emerge cirrhosis and hepatocellular carcinoma (liver cancer) like late complexities [4]. In contradiction to other cancers, the proportion of hepatocellular carcinoma precedes by 3-9% each year and is termed as the third root cause of cancer-related morbidities. It is noteworthy to detain liver markers such as aspartate aminotransferase (AST), alanine aminotransferase (ALT), and alkaline phosphatase (ALP) to clinically spot viral hepatitis and other types of liver disorders and hepatic flogs. The discretion of biochemical blood tests for enzymes is eminent for the clinical administration of this disease [5]. Similarly, there are other obstructions to cope with this disease like oxidative stress and hormonal imbalances. A build-up connection exists between endocrine frames and the liver where protein-binding hormones' production and inactivation crop up. Homeostasis of endocrine regularity may prompt to trouble hepatocyte function impairment. Furthermore, patients with hepatitis C have impaired regenerative endowment of fertility, weakness of men regenerative hormones, reduction of serum testosterone, abnormal values of luteinizing hormones and follicle-stimulating hormones, hypothalamic-pituitary malady, changes in spermatogenesis, and DNA variations (Hamid et al. [6]; [7, 8]). Different studies document that viral nucleocapsid protein plays a crucial role to boost up the reactive species in the liver cell thereby disturbing the host antioxidant system. Additional testing evaluated that antioxidant enzymes' levels including superoxide dismutase, peroxidase, catalase, and glutathione reductase are declined in individuals chronically infected with HCVs [9].

For a decade, scientists' extensive efforts had been undertaken until HCV's discovery and isolation. This disease is rarely fulminant and progresses to a substantial burden of associated morbidities. The first course of treatment was interferon monotherapy preceding virus discovery [4]. This medication was not quite effective and was associated with negative repercussions. Numerous approaches rectified the overall efficacy, viz., the addition RBV, the development of pegylated interferons (PEG IFN-*α*), and DAAs [10]. Onwards 1997, a weekly infusion of PEG-IFN-*α* coupled with RBV rectified the therapeutic effectiveness and cure rate. About 40-50% of HCV-infected individuals have been reported with sustained virological response (SVR) with the coadministration of both RBV and Peg IFN-*α*. This amalgamation was known as standard medication until 2013 for all genotypes [11]. The majority of the above medicaments are controverted owing to their extreme reactions. Over a long period of time, RBV along with Peg-IFN was commonly employed to cure hepatitis C. Administration of a long-time interferon therapy has obligated the credibility and prosperity of drugs lasting from 24 to 72 weeks; however, other selective observations were the target to treat HCV patients with the least severe side effects [12].Coadministration drug therapy with PEG-IFN-*α* and RBV is objectionable due to safety considerations, scanty tolerability, and its parenteral route of administration [13]. Concern for the implications of PEG-IFN-*α* and RBV on development and growth in this age class also limits their usage [14]. As such, recent international guidelines advocate that in patients over 12 years of age, treatment should be adjourned until DAAs are available [15, 16].

DAAs have been reported to extensively improve the treatment of hepatitis C. These drugs pose a potential efficacy in contradiction to the conventional treatment. Exquisitely, treatment of HCV disease is by all accounts simple with these new antiviral medications. An amalgamation of these direct acting drugs manifests a special impediment to resistance, elevating the cure rate [17]. A major step forward was the licensing of DAAs comprising triple therapies [18, 19], whereas the potential of treatment was steadily intensified, and safety concerns adequately became a constraining factor for utilizing this therapy. Thus, the swiftness of the development of new treatment regimens has considerably enhanced. Similar to the dual-drug treatment, the triple concomitant medication with first-generation DDAs poses adverse side effects [20].

The foremost substantiation of the concept of an interferon-free therapy was provided in 2010. Thus, SOF (Sovaldi®), a unique nuc-polymerase inhibitor, has been licensed in Canada, Europe, and the USA. This nuc-polymerase inhibitor is presumed to be the most effective of the presently available DAAs [21]. SOF is a nucleotide analog used for the treatment of HCV [22]. SOF manifests structural analogy with the substrate of RNA polymerase and binds its catalytic site; consequently, replication of HCV obstructs [23]. It is a medicine, which is turned into its metabolite tri-phosphate and becomes beneficial to cure infection [24]. It can be used as solitary or as an adjunct with other drugs such as RBV and ledipasvir [25]. SOF-built treatment has been dammed up to the major medications rundown of the World Health Organization (WHO), and probably it may corroborate to improve contagion or disease in a very short period. SOF and RBV combination has been reported to enhance the SVR rate up to 90%, but it also exhibits indirect consequences such as cerebral pain, rash, a sleeping disorder, weariness, queasiness, and iron dearth. Additionally, this therapy also seems to cause serious other side effects, for instance, angina pectoris and urethral damage in patients. Administration of SOF and RBV combination therapy provokes alternation in hematological parameters, especially a rapid drop on platelet count, which led to discontinuation of drug therapy in several patients [25]. RBV along with SOF intensifies the levels of AST and ALT in the rat model. SOF, as a solo drug or in combination with other drugs like RBV, has demonstrated to increase bilirubin levels. RBV also augmented bilirubin concentrations and caused anemia [25]. So far, it has been observed that ribavirin and sofosbuvir combination eradicates viral load expeditiously by ameliorating SVR to the maximum. However, there is a lack of knowledge regarding the side effects of these drugs on the regulation of pituitary and sex hormone levels and electrolyte balance in HCV patients [25].

Though antivirals effectively eradicate viral load, but these may also pose several other side effects. To revoke the side effects of antivirals, several ancillary supplements or adjuvant therapies are being used to treat HCV [26, 27]. Adjuvant therapy is supposed to enhance the patient's well-being and to restore the hepatic function and other parameters of viability. Literature shows that several vitamins and minerals such as A, B, C, E, D, zinc, and selenium are used as adjunct to ameliorate the various viability markers of the liver and blood in HCV [26, 27]. In this study, we managed to probe the effects of silymarin as an adjunct therapy in HCV patients along with SOF and RBV.

Silymarin is presumed to be a blend of flavonolignans or phytochemicals removed from the seeds and fruits of the plant named *Silybum marianum*. It is actually an extract of *Silybum marianum* which exhibits a profile of flavonoids, almost 20% to 35% of polyphenolic compounds and unsaturated fats, and one of its paramount isomer is silybin. Silymarin comprises of three phytochemicals: silidianin, silicristin, and silybin. It has a long historical tradition as a complementary and alternative medicine or herbal remedy. Silybin is considered as the most active and potential phytochemical and is largely supposed to be responsible for the proclaimed benefits of silymarin. Silymarin has been reported to have several pharmacological effects such as antiretroviral, antioxidant, anticancer [28], anticarcinogenic, immunomodulatory, cardioprotective, hepatoprotective, and anti-inflammatory effects [29–31]. Impediment of apoptosis associated with cellular death and inflammatory dynamization by silymarin exhibits its neuroprotective effect [32]. It has been remarked by clinical trials that silymarin is sound safe at higher doses (>1500 mg/day) in humans. Silymarin has now utilization as standard modern pharmaceutical drug akin to other prescribed drugs. The standard therapeutics in hepatitis C treatment evoke adverse side effects, such as relapse of this disease due to viral genetic diversity, drug resistance, anomalous hematological, and serological parameters, which are quite difficult to endure. Therefore, the establishment of new treatment modalities or adjunct therapies is expedient. Thus, the present miniature clinical study was intended to explore the potential effects of silymarin as an adjunct therapy along with SOF and RBV standard therapy. The prospective therapeutic effects of this adjunct therapy in our study were to probe the antiviral effects and to scrutinize the robust role of adjunct in ameliorating hematological, serological, and antioxidant markers.

## 2. Materials and Methods

### 2.1. Patients' Selection

Medical record of patients with primary diagnosis of hepatitis C infection was collected from Bakhtawar Amin Medical College and Hospital, Multan, Pakistan. The study was conducted in accordance with the Declaration of Helsinki and was approved by the Research Ethics Committee of the Department of Biochemistry, Bahauddin Zakariya University, Multan, and Bakhtawar Amin Memorial Hospital and Medical College, Multan (medical trial approval/2017/204), and consent form of each patient was obtained.

All patients were screened for hepatitis C virus infection and other similar infections like HBV (hepatitis B virus) and HIV (human immunodeficiency virus infection) infections. The patients were examined by gastroenterologist and hepatologist longitudinally. The inclusion criteria were followed by recruiting patients of both sexes who were of 18–50 years of age and were found positive for HCV infection. All other patients were excluded from the study. Exclusion criteria comprised patients with the history of any anti-HCV treatment of PEG-INF and RBV less than 6 months earlier to enrolment. Patients who were coinfected with HBV and HIV, as well as those who had any other illnesses or a history of cancer treatment prior to enrollment, were also excluded. Patients consuming alcoholic drink or any other antiviral medications, antenatal and lactating mothers, any sort of malignancies, and other ailments such as congestive heart failure, renal failure, respiratory failure, autoimmune diseases, and noncompliance to specific treatments were also excluded.

### 2.2. Study Design

A total of 30 HCV-positive patients participated in the study. The patients were divided into two groups.

- Group 1 (control group, *n* = 15) received SOF and RBV at a final dose of 400 and 800 mg per day as treatment, without any adjuvant therapy for a period of 8 weeks
- Group 2 (treated group, *n* = 15) received SOF and RBV 400 and 800 mg per day along with silymarin (400 mg per day) as adjuvant therapy for a period of 8 weeks. All the patients under study were monitored regularly to observe the effect of silymarin on the efficacy of SOF and RBV drugs when given in combination

### 2.3. Blood and Serum Specimen Collection

Venous blood samples (4 ml) from each patient of both groups were collected in EDTA (Atlas Labovac, K3 EDTA) and heparin (Sigma-Aldrich, Milan, Italy) vials using 5 ml syringe (Becton Dickinson, Singapore) for laboratory investigation. EDTA-treated blood was used for (cellular study) complete blood picture, while heparin-treated blood was used for plasma separation for biochemical analysis viral load and genotyping assessments. Heparinized blood was centrifuged at 6000 rpm using a centrifuge machine (EBA 20, Hettich-Centrifuge, Germany) to separate plasma, which was stored at -20°C for further biochemical analysis.

### 2.4. Complete Blood Count (CBC)

EDTA-treated blood was used for complete blood picture. A seven-part differential fully automated analyzer (Sysmex Japan) was used for CBC analysis. Hemoglobin (Hb) concentration, red blood cell (RBC) count, hematocrit-calculated absolute values, white blood cell (WBC) count, reticulocyte count, and differential leukocyte count were examined for all the participants.

### 2.5. Liver Function Tests (LFTs)

LFTs were conducted using Beckman Coulter (AU480, USA) to assess liver damage. The levels of ALP, AST, ALT, GGT, and total bilirubin were assessed and recorded.

### 2.6. Oxidative Markers

#### 2.6.1. Total Antioxidant Status (TAS)

TAS of each patient's serum was examined with a Randox reagents kit (Cayman Chemicals, USA), using UV spectrophotometer (Hitachi, Japan). Controls (pre- and posttreated samples) were run in parallel. The test assay covers the reaction of ABTS (2,2 ′ -azino-di-(3-ethyl-benzthiazoline-6-sulfonate)) with H_2_O_2_ and peroxidase (metmyoglobin) to generate the cationic radical (or ABTS+). Serum antioxidants proportionally minimize the concentration of ABTS+ that gives a quite stable blue-green color at 600 nm in a UV spectrophotometer. The results were expressed as U/l.

#### 2.6.2. Reduced and Oxidized Glutathione (GSH and GSSG)

Levels of reduced and oxidized GSH (L-*γ*-glutamyl-L-cysteinyl-glycine) in the patients' serum were quantified by Ellman's assay, where Ellman's reagent (5,5′-dithiobis(2-nitrobenzoic acid) (DTNB)) was used to react with GSH utilizing assay kit (by Cayman Chemicals, USA) on a microplate reader (Molecular Devices, Sunnyvale, Inc., CA). Blood was collected into a vacutainer tube (Becton Dickinson) containing EDTA. The hemoglobin (Hb) concentration was investigated with a hemocytometer, and 100 ml of the blood was immediately mixed with 12 ml of 10 mmol/l phosphate buffer, pH 7.2 (for GSH), and with 12 ml of 0.1 mol/l N-ethylmaleimide (NEM; for GSSG). A 100 ml aliquot of each mixture was hemolyzed by adding 900 ml of ice-cold distilled water. 100 ml of each hemolyzed sample was subjected to deproteinization by adding 200 ml of sulfosalicylic acid (120 ml/l), and the glutathione content in the acid-soluble fraction was determined. Known concentrations of GSH and GSSG at three different concentrations (100, 500, and 2500 mmol/l) were added to trial blood samples. The concentration in samples with added glutathione was determined in five replicates, and analytical recoveries were calculated. The intra-assay precision was obtained by analyzing 10 replicates of the biological samples in the same day. The intra-assay precision was determined by analyzing the same biological samples on 10 different days over 1 month. The CVs for repeatability of sample measurements were 0.5% for tGSH, 7.7% for GSSG, and 1.1% for GSH. The CVs for reproducibility, determined by assaying on 10 different days, were 2.8% for tGSH, 7.9% for GSSG, and 1.8% GSH. The mean recoveries were 89–102.2% for GSH and 96.1–114% for GSSG. Calibration curves for glutathione (5–100 mmol/l glutathione) were prepared in duplicate by diluting the stock solutions with 0.1 mol/l HCl containing 100 mmol/l DTT. The linearity of the assays was also assessed at glutathione concentrations of 0–100 mmol/l. The limit of detection for the calibrators is defined as the concentration that produces a signal-to-noise ratio. DTNB is converted into 2-nitro-5-mercapto-benzoic acid (TNB), which was quantified using a spectrophotometer with an absorption wavelength of 412 nm. This reaction examines the conversion of GSSG to GSH by a reduction reaction, envisaging the reaction rate proportional to GSSG and GSH concentrations. Ellman's reagent is a chemical used to quantify the number or concentration of thiol groups in a sample.

#### 2.6.3. Malondialdehyde (MDA) and Superoxide Dismutase (SOD)

MDA activity was analyzed on the basis of modified thiobarbituric acid utilizing specific TBA/TCA-specific reagents as described by the Janero method [33]. The SOD activity was assessed via the McCord and Fridovich procedure [34]. The principle of an antioxidant assay is based upon the determination of the antioxidant activity in the sample by defining their reaction with a specific concentration of exogenous H_2_O_2_. The antioxidants fairly exterminated a certain amount of available H_2_O_2_. The remaining H_2_O_2_ was quantified via colorimetric enzymatic reaction which involves the transformation of 3,5-dichloro-2-hydroxy benzene sulfonate to a specialized colored compound. Complete antioxidant activity was determined utilizing a TAC kit (Bio Diagnostics Inc.) and was analyzed using a spectrophotometer (Thermo Fisher Scientific, USA).

### 2.7. Sex Hormone Diagnostic Assay

The impact of silymarin adjuvant therapy was also determined on different sex hormones such as follicle-stimulating hormone (FSH), luteinizing hormones (LH), testosterone, and progesterone using the autoanalyzer (Cobas 6000 analyzer series). This assay functionality depends upon the electrochemical luminescence immunoassay (ELISA). Kit pack utilization is based on the principle of competitive binding among a chemical conjugated antigen and the serum specimen antibody for a steady measurement of immunological response. FSH and LH assessments were based on sandwich ELISA.

### 2.8. Viral Load Assessment

**Figure 1:**
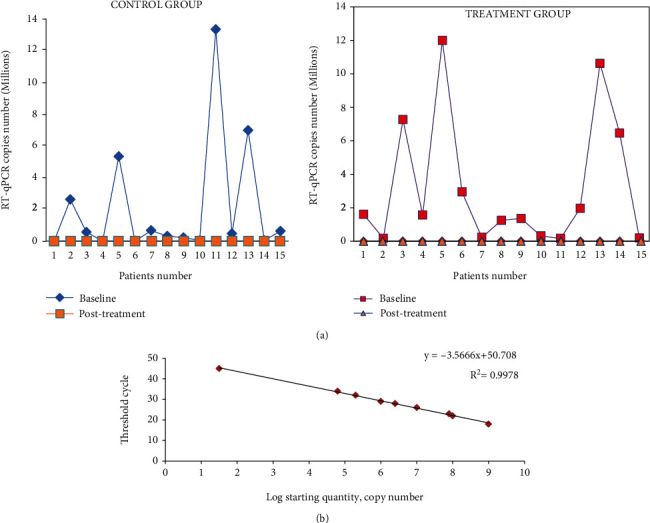
(a) Line plot of RT-qPCR levels in the control and treatment groups before and after adjunct therapy. (b) Standard curve generated by plotting the number of RNA copies vs. the corresponding RT-qPCR threshold cycle (Ct) value of three independent experiments.

Specified HCV RNA extraction was performed through an extraction kit (GF-1 Viral Nucleic Acid Extraction Kit, Vivantis, Inc., Malaysia). RT-PCR was done on the RT-PCR system (ABI 7500) utilizing the ROBO GENE-HCV RNA quantification kit for HCV with as much as lower detection limit of <50 copies. Real-time PCR is the most advanced technique used in in vitro diagnosis to detect the viral load of hepatitis C virus in the serum/plasma. HCV RNA was extracted from the sample, amplified by using the Real-Time Amplification and Detection kit having the florescent reporter dye (FAM) probes, specific for HCV 5′-UTR and specific primers for amplification. Negative and positive controls were used to maintain the quality. Eight quantitation standards were used to make the standard curve. The viral load of the sample was determined with the help of their threshold cycle and standard curve (Figure 1) having a cutoff value less than 200 copies/ml (Table 1). The titer of HCV in the blood fluctuates in accordance with the virus latency, and sometimes it may be absent or below the sensitivity limit of the assay.

### 2.9. Genotyping

The collected amplicons were primarily hybridized through oligonucleotide containing sequences specified for different HCV genotypes on nitrocellulose strip (GEN-C, RH Strips Assay, NLM, Inc., Italy). The bands for unique HCV genotype variants obtained through labeling of hybridized sequences with determined probes were examined to identify the specific genotypes.

### 2.10. Statistical Analysis

Statistical software (GraphPad Prism, Version 9.0) was utilized for the purpose of statistical analysis. One-way ANOVA test was used for *p* value determination, values were defined as the mean and standard deviation (mean ± SD), and *p* value less than 0.05 was considered significant.

## 3. Results

### 3.1. Hematological Parameters (CBC)

Our results showed that silymarin in combination with SOF and RBV significantly ameliorated blood parameters of treated patients as compared to the control group. SOF/RBV and silymarin adjunct therapy in the treated group triggered more production of RBCs (*p* value of 0.33), WBCs (*p* = 0.33), platelet count (*p* = 0.12), hemoglobin (*p* = 0.74), and neutrophils (*p* = 0.40) as compared to the control group (*p* > 0.05) (Table 2). The outcome of our results showed that silymarin adjuvant may have an ameliorative effect on the hematological parameters of HCV patients. A marvelous finding of adjuvant therapy was its boosting effect on platelet count (Table 2). Values were statistically nonsignificant within the groups themselves (*p* > 0.05).

### 3.2. Liver Function Markers (LFTs)

**Figure 2:**
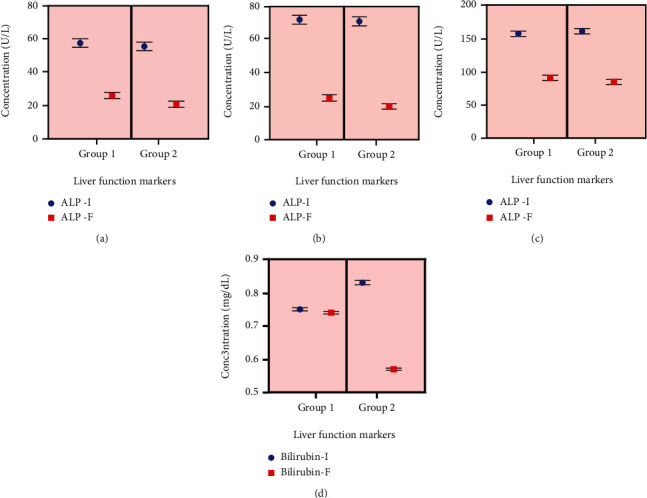
Effect of silymarin on liver function markers (a) AST, (b) ALT, (c) ALP, and (d) total bilirubin in group 1 (control group) and group 2 (treated group) at pre- and posttreatment level. Comparison was made by using one-way ANOVA test. Values were expressed as the mean ± SD. AST-I: initial aspartate aminotransferase; AST-F: final aspartate aminotransferase level; ALT-I: initial alanine aminotransferase level; ALT-F: final alanine aminotransferase; ALP-I: initial alkaline phosphatase; ALP-F: final alkaline phosphatase; Bilirubin-I: initial bilirubin level; Bilirubin-F: final bilirubin level.

Our results showed that combination of silymarin along with SOF/RBV in the treated group had a declining effect on the increased level of hepatic markers such as AST (Figure 2(a)), ALP (Figure 2(c)), ALT (Figure 2(b)), and total bilirubin (Figure 2(d)) as compared to the control group (*p* > 0.05). Values were expressed as the mean ± SD, *p* > 0.05 between the different groups (between treatment and control groups) and *p* > 0.05 within the groups among themselves (Figure 2).

### 3.3. Oxidative Markers

#### 3.3.1. TAS

**Figure 3:**
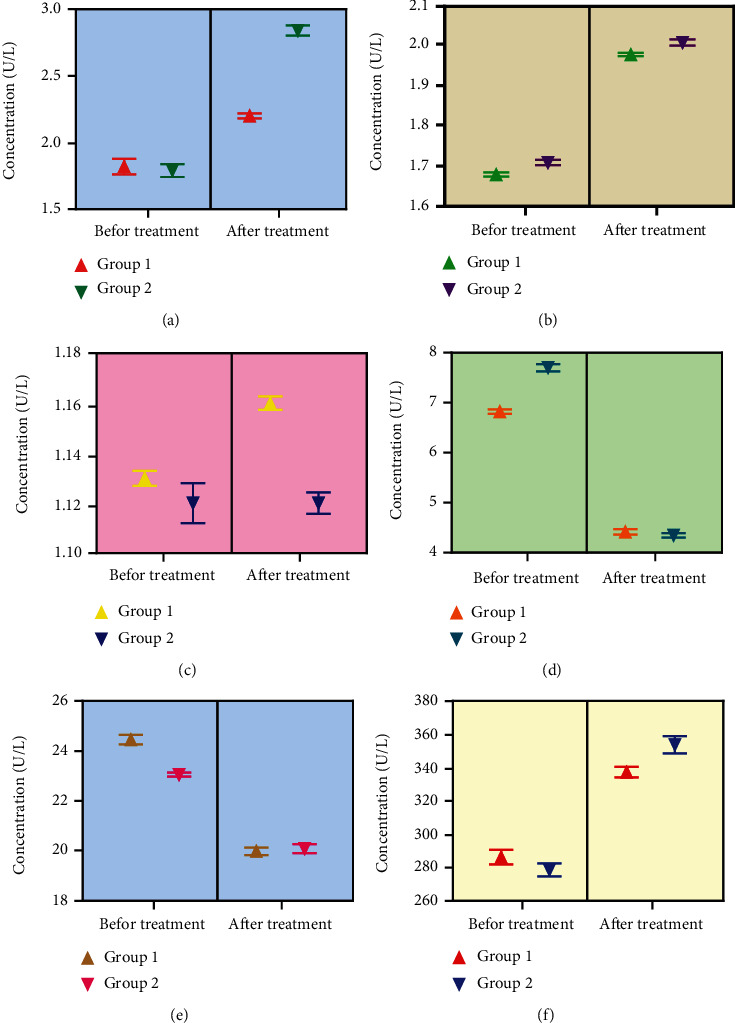
The level of (a) TAS, (b) GSH, (c) GSSG, (d) MDA (e) GGT, and (f) SOD oxidative stress markers in group 1 (control) and group 2 (treated group) at pre- and posttreatment level. Values were statistically nonsignificant at *p* > 0.05. One-way ANOVA was conducted to analyze the results. Data are expressed as the mean ± standard deviation. TAS: total antioxidant status; GSH: reduced glutathione; GSSG: oxidized glutathione; MDA: malondialdehyde; GGT: gamma-glutamyl transferase; SOD: superoxide dismutase.

Our posttreatment findings revealed that TAS level was found to be increased both in the control and treatment groups (SOF/RBV and silymarin+SOF/RBV). However, TAS was increased slightly higher in the treated group when compared to the control (*p* < 0.05) (Figure 3(a)).

#### 3.3.2. GSH

Our results demonstrated that GSH level was slightly increased after treatment both in the control and in the treated groups. However, the GSH level was found to be slightly higher in the treated group compared to the control group (*p* < 0.05) (Figure 3(b)).

#### 3.3.3. GSSG

Our research outcomes showed that the level of GSSG was declined after treatment in the both control and treated groups, but the GSSG level was more dwindled in the treated group over the control group (*p* < 0.05) (Figure 3(c)).

#### 3.3.4. GGT

Our results showed that the level of GGT was declined after treatment in both the control and treated groups, but the GGT level was decreased more in the treated group compared to the control group (*p* < 0.05) (Figure 3(e)).

#### 3.3.5. SOD and MDA

Our data showed an increasing effect on SOD level and a decreasing effect on MDA level in the posttreatment scenario in both the control and treated groups. However, silymarin adjunct therapy in the treated group indicated slightly more improved outcomes for the increasing effect on SOD and a decreasing effect on MDA when compared to the control (*p* < 0.05) (Figures 3(d) and 3(f)). However, for all oxidative parameters, values were statistically nonsignificant within the groups themselves (*p* > 0.05).

### 3.4. Sex Hormone Level

**Figure 4:**
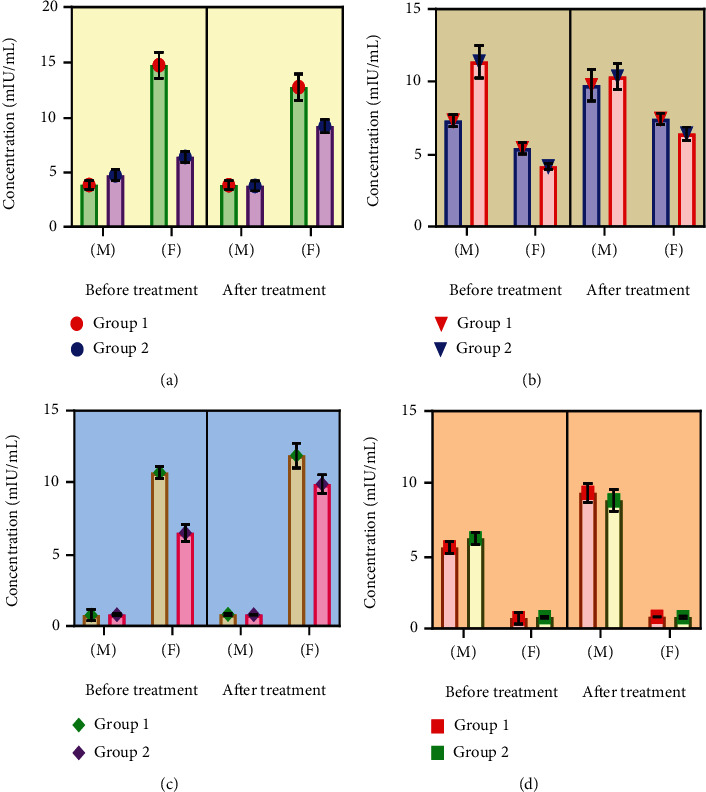
The level of (a) LH, (b) FSH, (c) progesterone, and (d) testosterone hormones in male and female control (group 1) and HCV-treated group (group 2), at pre- and posttreatment level. Values were statistically nonsignificant at *p* > 0.05. One-way ANOVA was conducted to analyze the results. Data are expressed as the mean ± standard deviation. M: male patients; F: female patients; LH: luteinizing hormone; FSH: follicle-stimulating hormone.

Our study showed that silymarin as an adjuvant therapeutic with SOF/RBV exhibited an ameliorating effect on hormonal level of both male and female HCV patients. In male patients, adjunct therapy tends to regulate LH (Figure 4(a)) and FSH level (Figure 4(b)) as compared to the control group in which administration of SOF/RBV (control) showed an increase in the level of LH and FSH (*p* > 0.05). Similarly, in the treatment group, this adjunct therapy tends to normalize the level of testosterone level by enhancing it (Figure 4(d)), when it was found to be declined in the control group (*p* > 0.05). It was noted that progesterone level remained almost constant in all groups, either the treated or the control group of males (*p* > 0.05) (Figure 4(c)). Values were statistically nonsignificant within the groups themselves (*p* > 0.05) (Figure 4).

Female patient's serum level of LH (Figure 4(a)) and FSH (Figure 4(b)) was probed to be increased in both the control and the treatment groups (*p* ≤ 0.05). This shows a beneficial or ameliorating effect of both drugs (SOF+RBV) and adjunct (SOF+RBV+silymarin). Silymarin tends to normalize the progesterone level in the treatment group that was found to be decreased in the control group (*p* ≤ 0.05) (Figure 4(c)), while testosterone level was demonstrated to be remained almost constant in females' group (Figure 4(d)). Values were statistically nonsignificant within the groups themselves (*p* > 0.05) (Figure 4).

### 3.5. HCV Detection by RT-qPCR

The findings illustrated that treatment of SOF/RBV alone in the control group and the adjunct treatment having SOF/RBV with silymarin in the treated group had utmost eminent same effect on inhibition of HCV replication and thus on the curtailment of viral load. Thus, in the both groups, SOF/RBV alone and adjunct therapy of SOF/RBV with silymarin removed the viral level well in all HCV patients.  Figure 1 and Table 3 correspond to the PCR outcomes at baseline and after treatment after duration of 8 weeks.

### 3.6. Genotyping

Genotyping performance manifests a significant trend of genotype 3a in both groups either the control or treated group with a slight frequency of 2b. Table 1 represents the results of genotyping.

## 4. Discussion

Hepatitis C is a problematic hepatic dysfunction caused by HCV [35]. The virus induces chronic liver infection, which is the main cause of fatality and morbidity globally [36]. Since the reporting of the virus in 1989, clinical trials have incited nonstop developments in diagnostic instruments along with administration approaches [37]. Previously, the combination therapy of RBV and peg-INF was suggested for HCV treatment. It relied on viral clearance in the immune system contrary to especially targeting the HCV with restricted acceptability and efficacy [36]. This combination therapy is the main cause of hematological aberration such as anemia, low platelet count, leucopenia, and thrombocytopenia in hepatitis C patients [38, 39]. Research has shown that SOF and RBV are among DAAs that effectively clear the virus and improve the SVR rate, but still their adverse effects subsist [40]. To overwhelm this challenge, our study probed the effects of silymarin (a plant-originated standard drug available at pharmacies) as an adjuvant therapy due to its antiviral, antibacterial, antioxidant, anticancer, immunomodulatory, and anti-inflammatory competencies [29–31]. Our study investigated whether silymarin in combination with SOF/RBV could ameliorate the efficiency of antivirals in HCV patients. Our data showed that silymarin as an adjunct therapy demonstrated an increasing effect on the level of Hb, WBC, RBC, PCV, and especially on platelet count in the treatment group as compared to the control. An intriguing finding regarding blood parameters was a boosting effect of silymarin adjuvant on platelet count, which has been reported to be plunged in HCV patients leading to quit of chemotherapy, while our data of solo therapy of DAAs did not display a remarkable effect on hematological parameters with a profound decline in platelet count. The results reported by Ahmadi et al. demonstrated that silymarin exhibits a lucrative effect on Hb and RBC in juvenile rainbow trout after a 4-week treatment [41]. Research has shown that silymarin therapy had a boosting effect on platelet count with 100 mg/kg dose per day in rats' model while combating nickel-based-induced toxicity [42]. Karimi et al. evaluated that silymarin has a beneficial effect on neutrophils, lymphocytes, and WBCs [43]. Another research report indicated that in albino rats, silymarin considerably improves the eosinophils and lymphocytes' level in cisplatin-induced toxicity [44]. These studies show consistency with our results; however, these were performed in animal models with different ailments. Thus, we can hypothesize that silymarin therapy may ameliorate blood parameters in hepatitis C patients and hence can be used to treat hematological abnormalities imposed by SOF and RBV drugs, especially a drop in platelet count in hepatitis C patients. Based on our preliminary results, the practitioners in our hospital started to give silymarin as an adjuvant therapy to hepatitis C patients with their own consent for better performance of treatment.

Our serological findings revealed that a higher level of ALT, ALP, and AST (U/l) substantially decreased to the optimum level in the treatment group. Conversely, SOF/RBV combination therapy was found to decrease the level of these enzymes in the control group after the treatment. Our results demonstrate that silymarin significantly enhances the level of these enzymes to improve the efficacy of standard SOF/RBV in the treatment group. The concentration of total bilirubin (mg/dl) was found to be substantially reduced to the normal level in the treatment group as compared to the control group despite *p* value is >0.05. Previous research has reported relative efficacy of SOF and RBV combination therapy in which ALT, AST, and other liver function markers were lowered, and bilirubin level was enhanced slightly after 12 weeks of therapy [45]. Our study revealed that silymarin as an adjuvant had an ameliorative effect on liver function enzymes, thus inhibiting the adverse effects of SOF/RBV on total bilirubin level and other liver function enzymes (Figure 2). A recent study has shown that silymarin provokes hepatoprotective effects by impeding the rising level of bilirubin and liver enzymes in male Wistar rats [46]. Another research has indicated the preventive effects of silymarin on liver function enzymes in rat's liver injury induced by carbon tetrachloride toxicity [47].

Oxidative stress leads to progression and development of hepatic impairment [48]. Oxidative stress and lower level of antioxidants may lead to chronic hepatitis [49, 50]. HCV may provoke cell damage and has been remained unclear. However, research inferred clearly that oxidative stress betrays a pathogenic role in chronic disease of HCV [51]. TNF-*α*, a prime cytokine, is thought to be responsible to trigger extreme oxidative stress through the generation of potential reactive oxygen ROS [51]. In the cell, a nonenzymatic antioxidant such as glutathione (GSH) plays a major critical role in the defense system against oxidative stress caused by infection. The reduced form of GSH can be transformed into an oxidized glutathione (GSSG) with the help of glutathione peroxidase (GSH-Px), which subsequently reacts with glutathione reductase to produce reduced form again. Cells also own their antioxidant mechanisms which perform a key role in the eradication of hazardous free radicals [52]. The flair enzymatic antioxidant defensive system in humans may contain GSH-Px, CAT, and SOD. It has been reported that SOD exhibits a latent capacity to the cells from toxicological impact of free superoxide radicals [52]. GSH-Px decays hydrogen peroxide via transforming lipid peroxides to nontoxic molecules, thus preventing the cells from the detrimental effects of lipid peroxidation [53]. Our findings report that both SOF/RBV and silymarin adjunct therapy showed an enhancing effect on TAS, SOD, and GSH level, while depleting the level of GSSG, MDA, and GGT. However, silymarin therapy's results were slightly more promising than SOF/RBV alone. A research has declared that anti-HCV therapies in patients may boost level of TAS, SOD, and GSH and may decline the level of MDA, GSSG, and GGT [51]. It has been declared that the intensity of oxidative markers such as MDA is related to the severity level of chronic hepatitis C [54]. Our results are supported by previous reports that silymarin may counterbalance the oxidative stress and reinforce the potential human antioxidant system [55–57]. Thus, our data suggest that silymarin can be utilized as a potential antioxidant supplement to subside HCV (Figures 3(a)–3(f)).

The paradigm of deference in the pathogenesis of genotypes has been remained an obscure. But the genotype is assumed to be as one of the major predictors of HCV regarding antiviral therapies. Owing to genotypic specific variation in response to the new generations of antivirals, HCV genotype estimation may facilitate the better management of proper strategies, especially during treatment therapy [58]. Thus, as our study shows, antioxidant supplementation in HCV patients with prime-resistant genotypes may provide better results. Research manifests that vitamins E and C and selenium supplements as adjuvants may boost the antioxidant activity with no quite prominent impact on the viral load [59]. Thus, antioxidants' effect on the viral load and SRV could be the subject matter of future investigations. Intriguingly, our study demonstrates that SOF/RBV therapy may ameliorate oxidative markers, but these are not typical or specialized antioxidants. Their antiviral efficacy might be supposed to suppress the inflammation and viral load, and thus, possibly this strategy may be responsible to decrease virus-induced oxidative stress, a probable mechanism as noted in our research study for reducing noxious oxidative stress. Hence, our findings reveal that antioxidants such as silymarin may be offered as an adjuvant with other regular antivirals to improve HCV pathogenicity.

A build-up connection exists between endocrine framework and liver function, where protein-binding hormones' production and inactivation of hormones have been reported to work together. Endocrine system homeostasis may lead to inconvenience of hepatocytic function in pathogenesis. Immune system regulates several reproductive cycles, so deviations from standard immune responses might affect fertility [60]. A research report has concluded the dysregulation of sex hormones in HCV patients [61]. Our investigation's serological findings disclose that silymarin as an adjuvant had a remolding impact on LH, FSH, progesterone, and testosterone level (U/l), which was observed to be declined significantly to normal in the treated group in contrast to the control (Figures 4(a)–4(d)). According to the literature, silymarin has exceptional effects on fertility regulation [62]. By assuming antioxidant action of silymarin and evaluating the impact of silibinin, which is the most effective flavonoid of silymarin, a significant improvement in testosterone and testicular-related factor was observed [63]. The results of our investigation are upheld by previous investigations conducted in animal models, which reveal that silymarin is by all means adequately effective to regulate the level of LH, FSH, gonadotropin-releasing hormone, testosterone, and fertility [64–66].

In our study, HCV possessed viral RNA was quantified before and subsequent therapy; surprisingly, the viral load of both groups was out of detection threshold after an 8-week therapy. Treatment with SOF/RBV effectively diminished the viral RNA from infected patients. At this point, we were unable to estimate the effects of silymarin adjunct therapy on viral quantification because viral load was reduced in both groups, indicating no comparison. Thus, our results also indicate that new generation of DAAs and SOF/RBV are enough to eliminate HCV viral RNA (Figures 1(a) and 1(b)). Zeuzem et al. reported the coherent effects that patients treated with DAAs can achieve SVR rate followed by a 4-week therapy [67]. Although this study did not elaborate on the effect of adjunct therapy on viral RNA quantification, we can assume that silymarin adjunct therapy may aid in viral load elimination, which has been well supported by other studies in support of our findings [68]. It has been stated that regular administration of silibinin eradicated the virus by 3 to 4 logs [69]. Furthermore, the effect of silymarin on the removal of the virus should be addressed over a shorter time period or at intervals to investigate its role in actual rectification. Overall, silymarin, if probed for antiviral potentiality, may play a role of analog to other drugs that regulate this disease by altering different parameters or markers involved at the serological and hematological levels through an antioxidant bridge, as we observed in our study. We may speculate that administration of silymarin may exhibit a potential role in the progression of HCV infection that is ultimately attributable to its immunomodulatory, antioxidant, antiviral, antimutagenic, and anti-inflammatory effects [55–57].

## 5. Conclusion

Our study infers that the systematic administration of silymarin as an adjunct therapy results in substantial amelioration of hematological parameters, especially platelet count. Our data showed that silymarin as an adjunct therapy demonstrated an increasing effect on the level of Hb, WBC, RBC, and PCV. It also has an indirect effect on the immunological and antioxidant systems. Silymarin may boost level of TAS, SOD, and GSH while decrease the level of MDA, GSSG, and GGT. It has been shown to effectively regularize liver function markers (AST, ALP, ALT, and bilirubin) and sex hormones (LH, FSH, progesterone, and testosterone) and thus may be able to suppress the hostile effects of SOF/RBV by increasing or decreasing effects. Our study also reveals that single SOF/RBV treatments are absolutely efficient in eradicating viral load, albeit with some side effects at serological, hematological, and hormonal levels. We anticipate that our preliminary findings will hasten future research into whether silymarin can coexist with and/or replace immunoregulatory medications used in anti-HCV therapy. Future research studies with a large size of the samples are ultra-appealing and have a greater statistical power that may lay the basis to reaffirm our contemporary results.

## Figures and Tables

**Table 1:** Genotyping frequencies of HCV-positive patients in the control and treatment groups.

Groups	HCV genotype	Frequency (%)
Control	3a	14 (94.23)
Treatment	3a	13 (86.66)
Control	2b	1 (0.066)
Treatment	2b	2 (1.33)

**Table 2:** Effect of silymarin and SOF/RBV adjunct therapy on hematological parameters of selected HCV patients.

Parameters	Pretreatment		*p* value	Posttreatment		*p* value
	Control	Treated		Control	Treated	
RBC (10^12^/l)	5.86 ± 8.86	5.29 ± 0.69	0.386	4.30 ± 0.34	6.43 ± 8.92	0.335
WBC (10^9^/l)	8.26 ± 1.75	7.88 ± 3.35	0.627	6.62 ± 2.11	9.88 ± 2.03	0.337
Hb (g/dl)	12.92 ± 1.43	13.35 ± 1.39	0.533	11.53 ± 1.33	14.16 ± 2.32	0.74
PCV (%)	42.12 ± 4.12	40.01 ± 7.08	0.672	41.66 ± 14.37	43.20 ± 15.98	0.005
MCV (fl)	80.55 ± 16.62	100.05 ± 7.51	0.673	75.47 ± 21.34	99.08 ± 5.12	0.01
MCH (pg)	28.62 ± 5.14	25.60 ± 2.36	0.022	27.47 ± 3.75	26.68 ± 3.55	0.001
MCHC (g/l)	33.23 ± 0.75	30.90 ± 0.58	0.248	32.46 ± 1.27	30.99 ± 1.34	0.991
Lymphocytes (%)	3.18 ± 5.52	31.06 ± 7.64	0.661	31.0 ± 10.48	31.40 ± 8.38	0.003
Monocytes (%)	2.2 ± 0.77	2.48 ± 0.34	0.166	3.1 ± 0.74	2.82 ± 0.63	0.695
Eosinophils (%)	3.01 ± 0.25	3.33 ± 1.54	0.526	3.73 ± 1.12	3.93 ± 1.16	0.008
Platelets (10^9^/l)	246.6 ± 55.10	257.6 ± 59.73	0.467	220.73 ± 46.53	279.66 ± 83.01	0.123
Neutrophils (%)	64.76 ± 6.08	65.0 ± 9.15	0.642	62.0 ± 9.28	66.33 ± 11.95	0.401

Data values were expressed as the mean and standard deviation (mean ± SD).One-way ANOVA test was utilized for comparison between different groups. Values were statistically nonsignificant within the groups themselves (*p* > 0.05). CBC: complete blood count; RBC: red blood cells; Hb: hemoglobin; PCV: packed cell volume; MCV: mean corpuscular volume; MCH: mean corpuscular hemoglobin; MCHC: mean corpuscular hemoglobin concentration.

**Table 3:** PCR values in the control group and the treated group at baseline and posttreatment level.

No. of patients	Baseline/pretreatment value	Posttreatment CT value	Baseline	Posttreatment CT value(copies/ml)
1	3002.72	>200	1613494.26	>200
2	2613494.26	>200	179248.23	>200
3	563209.95	>200	7269586.57	>200
4	3002.72	>200	1568413.49	>200
5	5319070.54	>200	11993056.68	>200
6	4139.28	>200	2949439.93	>200
7	665580.14	>200	237541.19	>200
8	313401.93	>200	1256184.28	>200
9	217541.19	>200	1365580.14	>200
10	37541.19	>200	326375.11	>200
11	13311070.54	>200	175723.96	>200
12	465580.14	>200	1972071.82	>200
13	6968413.49	>200	10621104.28	>200
14	42326.77	>200	6460580.14	>200
15	624481.23	>200	199126.77	>200

CT value < 200 no. of copies = HCV positive (titer: above the sensitivity).CT value < 200 no. of copies = HCV negative (titer: below the sensitivity).

## Data Availability

Datasets generated during and/or analyzed for this study project have been included in the main text. Data pertaining to ethics and patients' privacy or any other supplementary materials/data are available from the corresponding authors upon reasonable request.
